# Precise visuotopic organization of the blind spot representation in primate V1

**DOI:** 10.1152/jn.00418.2014

**Published:** 2015-03-11

**Authors:** João C. B. Azzi, Ricardo Gattass, Bruss Lima, Juliana G. M. Soares, Mario Fiorani

**Affiliations:** Institute of Biophysics Carlos Chagas Filho, Federal University of Rio de Janeiro, Rio de Janeiro, Brazil

**Keywords:** striate cortex, optic disk, blind spot, filling in, perceptual completion

## Abstract

The optic disk is a region of the retina consisting mainly of ganglion cell axons and blood vessels, which generates a visual scotoma known as the blind spot (BS). Information present in the surroundings of the BS can be used to complete the missing information. However, the neuronal mechanisms underlying these perceptual phenomena are poorly understood. We investigate the topography of the BS representation (BSR) in cortical area V1 of the capuchin monkey, using single and multiple electrodes. Receptive fields (RFs) of neurons inside the BSR were investigated using two distinct automatic bias-free mapping methods. The first method (local mapping) consisted of randomly flashing small white squares. For the second mapping method (global mapping), we used a single long bar that moved in one of eight directions. The latter stimulus was capable of eliciting neuronal activity inside the BSR, possibly attributable to long-range surround activity taking place outside the borders of the BSR. Importantly, we found that the neuronal activity inside the BSR is organized topographically in a manner similar to that found in other portions of V1. On average, the RFs inside the BS were larger than those outside. However, no differences in orientation or direction tuning were found between the two regions. We propose that area V1 exhibits a continuous functional topographic map, which is not interrupted in the BSR, as expected by the distribution of photoreceptors in the retina. Thus V1 topography is better described as “visuotopic” rather than as a discontinuous “retinotopic” map.

blood vessels covering the retina obstruct portions of the visual scene from stimulating photoreceptors at the fundus of the eye (Adams and Horton 2002, 2003a, 2003b). Similarly, the optic disk, a region of the retina devoid of photoreceptors, generates a visual scotoma known as the blind spot (BS). Such discontinuities in the visual field are not usually perceived with monocular vision because of a rapid bottom-up visual representation reconstruction, a phenomenon known as perceptual completion (Fiorani Júnior et al. 1992). Bottom-up visual processing is considered the result of ascending projections acting through a series of hierarchical visual areas (Hubel and Wiesel 1968). In the primary visual cortex (V1), information from both eyes arrives segregated into layer 4C. Within the BS representation (BSR), layer 4C only receives projections from the ipsilateral temporal hemiretina (Adams et al. 2007; Rosa et al. 1988). At each stage, intrinsic or horizontal connections reinforce the interplay of information processing among groups of neurons with similar receptive field (RF) properties (Hubel and Wiesel 1974; McGuire et al. 1991a, 1991b; Rosa et al. 1992). Early investigations proposed that the intrinsic lateral connections are the anatomical substrate for RF surround properties, as observed using electrophysiological recordings, and are thus responsible for perceptual completion in V1 (Fiorani Júnior et al. 1992; Gattass et al. 1999; Gilbert and Wiesel 1992). Numerous studies have shown the involvement of early visual cortical areas but also of higher-order visual areas in providing top-down feedback to early visual cortex in these perceptual processes (De Weerd et al. 1995; Hochstein and Ahissar 2002; Lin and He 2012; Matsumoto and Komatsu 2005; Moratti et al. 2014). Nevertheless, the neural mechanisms of completion or filling in are far from being completely understood. Using electrophysiological recordings, Fiorani Júnior et al. (1992) observed an apparent topographic organization within the BSR in area V1. Visual stimulation was shown monocularly to the contralateral eye (CE) so that no direct visual input would arrive at the BSR. The topography within the BSR, which was constructed via mechanisms involving perceptual completion, was surprisingly well organized. The RFs in these experiments, however, were hand mapped and therefore lacked the necessary precision to establish any quantitative conclusion. Matsumoto and Komatsu (2005) also found neurons in the BSR of V1 that are activated by a long bar, but all these neurons had RFs that extended outside the BS. In the present study, using unbiased automated RF-mapping procedures (Fiorani et al. 2014), we systematically investigated and quantified the topographic organization (scatter) and RF properties (size, ocular dominance, orientation, and direction selectivity) of V1 neurons inside and outside the BSR in the capuchin monkey (*Cebus apella*). A preliminary account of this work was presented previously in abstract form (Azzi et al. 2000).

## MATERIALS AND METHODS

### 

#### Animals.

Three adult male *Cebus apella* monkeys (animals A, B, and C) were studied in once-weekly recording sessions using single electrodes, for four sessions. One additional monkey (animal D) was studied with multiple (32) electrodes for a single session. Animal D was also used in a previous work (Botelho et al. 2014). All experimental protocols were conducted following the National Institutes of Health (NIH) guidelines for animal research and were approved by the Committee for Animal Care and Use of the Institute of Biophysics Carlos Chagas Filho, Federal University of Rio de Janeiro.

#### Surgical procedures.

Before the recording sessions, a head bolt and a recording chamber were implanted on the skull of each monkey, under anesthesia and aseptic conditions. Before surgery, anesthesia was induced with 30 mg/kg of ketamine hydrochloride (Ketalar; Parke Davis, Rio de Janeiro, Brazil). In addition, animals also received 0.15 mg/kg of atropine sulfate (Atropina; Roche, São Paulo, Brazil) to reduce salivation and other secretions and 0.8 mg/kg of benzodiazepine (Valium, Roche) to induce sedation. The animals were intubated with an endotracheal tube, and anesthesia was maintained throughout the procedure with 2% halothane (Fluothane; AstraZeneca, São Paulo, Brazil) in a 7:3 mixture of nitrous oxide and oxygen. We positioned the recording chamber to enable access to area V1, using stereotaxic coordinates and the position of the cortical sulci. Electrocardiogram, body temperature, and end-tidal CO_2_ were monitored continuously to ensure anesthesia depth and animal wellbeing during surgery. Additionally, we administered postsurgical analgesia for 3 days using a fentanyl skin patch (Durogesic; Janssen-Cilag, São Paulo, Brazil). The animals were monitored for a few days after surgery to ensure their wellbeing and prompt recovery.

#### Recording sessions.

For recording sessions, the animals were initially anesthetized with 30 mg/kg of 5% ketamine hydrochloride solution (Ketalar), intubated with an endotracheal tube, and maintained during recording with a gaseous mixture of 70% nitrous oxide and 30% oxygen, combined with a continuous intravenous infusion of fentanyl citrate (0.003 mg/kg per h). The monkeys were immobilized with pancuronium bromide (0.1 mg/kg per h iv) and assisted by a pressure-controlled ventilation unit (55-0798 ventilator; Harvard Apparatus, Holliston, MA). Electrocardiogram, body temperature, and end-tidal CO_2_ were monitored continuously to ensure anesthesia depth and animal wellbeing during the procedure. Postexperimental analgesia was administered for 24 h with a fentanyl skin patch (Durogesic). Animals were monitored to ensure their complete recovery from the anesthesia and recording session.

Gas-permeable contact lenses were used to focus the eyes on a 21″ screen monitor (model JC-2002 VMA CRT; NEC, Tokyo, Japan) placed 57.3 cm in front of the animals’ eyes. The positions of the BS and fovea were plotted onto the computer screen with a 180° reversible ophthalmoscope. Throughout the experimental session, we plotted the borders of the BS and tested the responsiveness of the neuron to small stimuli delivered through each eye in turn to assure the positioning of the electrode inside the BSR.

**Fig. 1.**
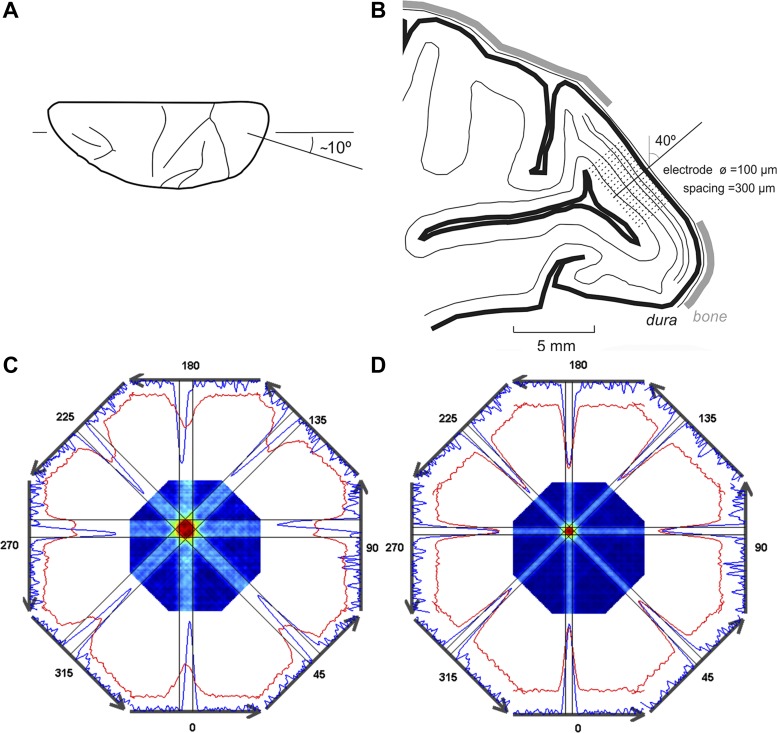
Electrode positioning and receptive field (RF) mapping. *A*: electrode penetrated the blind spot representation (BSR) at an angle orthogonal to the cortical surface. Shown is the top view of a monkey brain (left hemisphere) illustrating the medial-lateral tilt (∼10° lateral) of the electrode penetration along the horizontal plane. *B*: parasagittal section illustrating the ∼40° posterior tilt of the electrode penetration. Note that the BSR is located on the calcarine region, which is situated below the operculum. *C* and *D*: intersected maps (i.e., RF maps) obtained through the global mapping method. Surrounding the intersected maps are the corresponding spike-density functions (blue lines) and the interpolated RF profiles (red lines) for the 8 directions of stimulus motion (dark gray arrows) that were used to generate the maps. Thin black lines depict the RF limits (75% of peak amplitude). Intersected map without latency correction (*C*) and with a latency correction of 80 ms (*D*). Note that, without latency correction, the RF map is bigger and has a complex profile (donut shape) compared with the latency-corrected map. Additionally, the RF profiles (red lines) are broader and exhibit a lower average amplitude. The RF position (i.e., RF center) and RF size thus estimated were used for subsequent analyses.

To locate the portion of V1 corresponding to the BSR, we searched the cortex of the calcarine fissure at 1-mm intervals with a 1-MΩ impedance tungsten microelectrode, using stereotaxic coordinates and sulcal landmarks (Gattass et al. 1987; Rosa et al. 1988). The corresponding V1 stereotaxic coordinates were saved to allow access to the BSR in subsequent recording sessions. The microelectrode was carefully angled so that it would penetrate the calcarine cortex orthogonal to its surface, along a cortical column (see Fig. 1, *A* and *B*, for the specific penetration angles). We monitored the transition from the opercular surface to the roof of the calcarine sulcus by advancing the microelectrode in 200-μm steps. We stopped advancing the microelectrode when the first transition from a central RF (typically ∼5° eccentricity) to a peripheral RF (14–18° eccentricity) was observed. At this point, we allowed at least 10 min for electrode stabilization. RFs were initially mapped for every site using a manual procedure. We aimed at obtaining a sequence of RFs that started outside the BS border and that crossed the BS approximately along the horizontal meridian representation (e.g., see the RF sequence in Fig. 4). To ascertain that the electrode was inside the BSR in V1, no neuronal activity could be elicited for the following two conditions: *1*) stimulating exclusively inside the BS with moving bars using CE stimulation and *2*) stimulating exclusively inside the BS with small flashed squares using CE stimulation. These two steps were carried out for all monkeys studied before running the automated mapping protocols (see below). Throughout the text, we will use the term BSR to designate the cortical region of V1 corresponding to the optic disk of the CE. Note, however, that the same BSR also corresponds to a region of the ipsilateral eye (IE), which typically contains photoreceptors. Therefore, when we state that we are investigating the topographic organization of the BSR, we actually mean that we are studying the RFs mapped using monocular visual stimulation of the CE. Throughout the text, we also refer to regions outside the BSR. In this case, we refer either to cortical regions strictly outside the BSR or to the BSR itself when stimulating through the IE. This is because we here assume that the topographic organization of the BSR using IE stimulation is equivalent or comparable to that found for the rest of V1. Indeed, our evaluation of the topographic organization of the BSR was based on systematic comparisons between RFs obtained with CE vs. IE stimulation. We favored this strategy because comparisons could be made for the same recording and not across recordings, as would be the case, for example, when comparing RFs inside and outside the BSR for CE stimulation only.

We typically acquired neuronal data at two different depths (i.e., 2 sites) for each penetration in the calcarine cortex. The first site was located where we first obtained a stable recording (presumably in the infragranular layers). The second site was located after advancing the electrode 500–600 μm relative to the first site (presumably in the granular or supragranular layers). Neuronal data were acquired in blocks of 10–15 trials for each eye separately using the global mapping method. Electrode activity was amplified using an A-M recording system (model 1800; A-M Systems, Carlsborg, WA) and filtered (300-10,000-Hz band-pass filter) using a Krohn-Hite 3500 (Brockton, MA). For each site, we acquired two recordings: a well isolated single unit (SU) and a multiunit activity (MU). Both were sampled at 192 KHz using a waveform discriminator system (SPS-8701; Signal Processing System, Malvern, Victoria, Australia). The CORTEX software (Laboratory of Neuropsychology, NIMH/NIH, Bethesda, MD) was used for visual stimulation and to store extracellular spike events.

A major concern in our study was drifts in eye position taking place along the session because this could compromise the precision of our RF mapping relative to the estimated BS position. As a control, we thereby inserted an extra electrode at the opercular surface of V1 and mapped its RF position throughout the session. In general, no significant drifts were observed, which indicated that eye position was stable during the recording stage.

In animal D, we used an array of 32 tungsten microelectrodes (6 × 6 arrangement, where the electrodes at each of the 4 corners were absent). Each electrode had an impedance of ∼1 MΩ at 1 kHz and was separated by 0.7 mm from its nearest neighbor. All multielectrode data were acquired during a single experimental session and a single array placement. Before inserting the array, we probed the calcarine cortex using a single electrode to determine the approximate location of the BSR. The dura mater covering the corresponding region of the operculum was removed to avoid compression and facilitate the entrance of the array in the cortex. Finally, we slowly advanced the array through the operculum and then into the calcarine cortex, allowing us to record MU activity at several depths. The signals were amplified and filtered between 0.7 and 5.9 kHz (HST/16o25 headset, 32-channel preamplifier box; Plexon, Dallas, TX) before being digitized at 32 kHz by a high-speed, 16-bit resolution A/D card (PCI-6259; National Instruments, Austin, TX). Signal display, acquisition, and storage were controlled by a custom-written software (SPASS), kindly provided by Dr. Sergio Neuenschwander (Max-Planck Institute, Frankfurt, Germany).

#### Data analysis.

For signal conditioning, cortical neuronal responses usually show a high trial-by-trial variability. Responses were thereby averaged over 10 trials for each condition (stimulus direction) and binned (averaged) in 10-ms segments. To further smooth the signal, we convoluted each spike with a 60-ms (6 bins) Gaussian function (area equal to 1), converting the response histogram to a spike-density function (SDF). To compare neurons exhibiting different levels of spontaneous and driven activity, firing rates as expressed in the SDF were normalized to *z*-score units. The conversion was done by first subtracting the mean spontaneous activity (averaged across all trials) from the stimulus-driven activity. The resulting SDFs were then divided by the standard deviation of the entire signal. The shapes of the SDF and the *z*-score-normalized curves were the same but with a different abscise scale, now representing the normalized neuronal responses in SD units relative to the average of the entire signal.

#### RF mappings.

We employed a local and a global mapping method to investigate the short- and the long-range mechanisms, respectively, contributing to the topographic organization of the BSR. Both are fully automated mapping procedures providing a bias-free estimate of both RF position and size.

For global mapping, we used the methodology developed by Fiorani et al. (2014), which has been successfully employed in several previous investigations (Botelho et al. 2014; Jansen-Amorim et al. 2011, 2012; Soares et al. 2004). This method is based on computing the latency-corrected neuronal activity in response to white (50 cd/m^2^ luminance), elongated (30° × 0.3°) bars crossing the BS. The rationale was that neuronal activity generated at the surround of the BSR by the flanks of the elongated bar would give rise to activity within the BSR by means of previously described completion mechanisms (Fiorani Júnior et al. 1992). The bars were presented on a neutral background (10 cd/m^2^ luminance) and moved across the screen at a speed of 10°/s (total excursion, 30°) in eight different directions (0°, 45°, 90°, … 315°).

Latency imposes a delay in the neuronal response that needs to be corrected for to precisely estimate RF center and borders. We estimated the latency of each neuron by means of a heuristic method in which 10 empirical values, equally spaced between 20 and 120 ms, were tested. For each latency value, we created an intersected map using the responses to the eight directions of stimulus motion (see Fig. 1, *C* and *D*, for examples of intersected maps). The value that led to the highest response peak, corresponding to the maximal coincidence in peak response across all directions, was selected as the correction value for the latency (Fig. 1*C*, without any latency correction; Fig. 1*D*, with a latency correction of 80 ms). The latencies we estimated in our data (∼60–120 ms) were comparable to those usually reported for V1 (Maunsell and Gibson 1992; Raiguel et al. 1989).

We also used a local mapping method, which was designed not to elicit the long-range completion mechanisms made possible with global mapping. The local mapping method is based on the reverse correlation of neuronal activity in response to small white squares (0.5° × 0.5° size, 50 cd/m^2^ luminance) presented in one of 900 possible locations within a 15° × 15° panel (background luminance: 10 cd/m^2^). Squares were continuously presented at different locations for 1/60 of a second within trials that were 15 s long. This timing enabled all 900 possible locations on the panel to be randomly sampled. Responses at each location were averaged across 30 trials to build a 2D array map (smoothed using a 2D convolution with a 2.5° Gaussian window; see Botelho et al. 2014). Data for the local mapping method were systematically stored only for animal D, thereby serving as a quantitative control to assure that the electrode was positioned inside the BSR. For animals A, B, and C, the same kind of local stimulation was performed, albeit manually, using squares and small bars presented solely inside the BS.

**Fig. 2.**
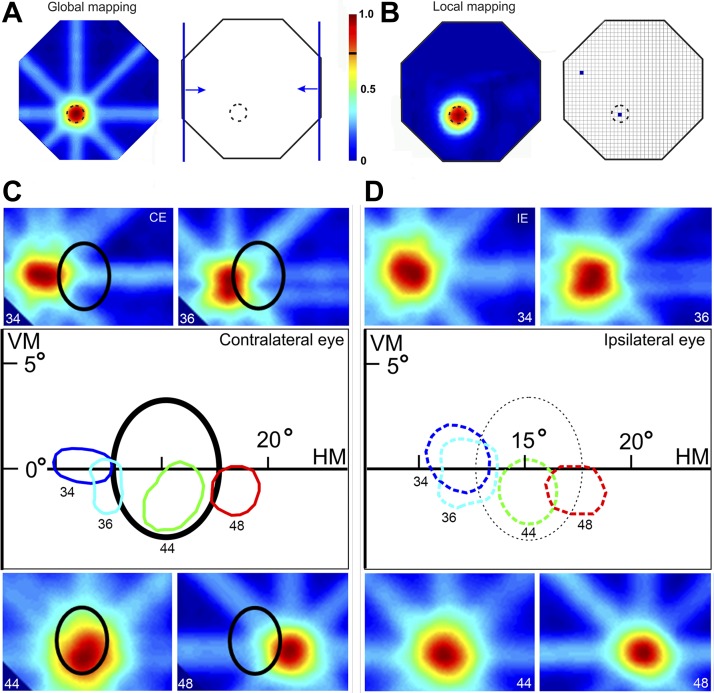
Examples of RFs mapped using the 2 automatic procedures. *A*: global mapping. *Left*: color-coded 2D representation of neuronal responses (intersection map) elicited by elongated bars moving in 8 different directions. *Right*: 2 conditions where the bar moves at 0° and 180°. *B*: local mapping. Color-coded 2D representation of the neuronal responses (*left*) using small flashed stimuli (*right*, small squares). RF borders (dashed lines), from which we extracted information regarding RF center and size, were estimated at 75% of the peak neuronal response. Note that the global mapping method generates artifacts on the RF map (elongated extensions around the RF), which do not compromise mapping per se. Responses are shown on a normalized scale (color bar between *A* and *B*, where the black mark indicates the 75% RF border threshold). *C*: RFs mapped with contralateral eye (CE) stimulation using the global mapping method (same as sites 34, 36, 44, and 48 illustrated in Fig. 4). VM, vertical meridian; HM, horizontal meridian. *D*: RFs obtained with the same mapping method and from the same recordings as those shown in *C* but using ipsilateral eye (IE) stimulation. Note the similarity between the RFs mapped with CE and IE stimulation despite the fact that only the IE sends direct visual input to the BSR in V1. The central panels in *C* and *D* depict the RF boundaries estimated from the corresponding RF maps. The oval shapes represent the BS. Note that the BS is present only for the CE at that location (continuous line). The dashed oval shape in *D* illustrates the location of the BS of the CE. The BS of the IE is located in the other hemifield, in a region not illustrated in this figure.

Figure 2, *A* and *B*, compares RFs obtained with the global and local mapping methods. The half-peak in the SDF for each of the eight directions of motion tested delineated a polygon, which corresponded to 50–75% of the net neuronal activity (Dow et al. 1981; Schiller et al. 1976). This polygon was taken as a proxy for our mapped RF. The automatic mapping procedures enabled us to extract quantitative information regarding both RF center and size. They were computed, respectively, as the center and square root of the area of the mapped polygon. The contours of all recorded RFs were superimposed, allowing direct comparison of their center and size, as well as their position relative to the projected edges of the optic disk (Fig. 2, *C* and *D*). As will be further explained in results, completion mechanisms generated by neuronal activity in the regions surrounding the BSR allowed us to map RFs that were largely contained inside the BS (e.g., the RF mapped for recording site 44 in Fig. 2*C*). To standardize data analysis and figures, we converted data for both left and right hemifields into a right hemifield representation.

#### Alignment of RFs obtained with IE and CE stimulation.

Because of neuromuscular blocking, the IE and CE were not necessarily aligned during the experiment. Therefore, sequences of RFs obtained outside the BS with IE and CE stimulation were superimposed and aligned by minimizing the average distance between their corresponding RF centers (i.e., minimum error). Therefore, with our RF alignment procedure, we aimed at minimizing mean disparity by assuming that the real mean disparity between RFs mapped with IE and CE stimulation was 0. Note that we did not interfere with the spatial relationship (i.e., scatter) between RFs mapped within each eye because they were jointly shifted during alignment.

#### Scatter analysis.

We used a modified measure of RF scatter to quantify the topographic organization of area V1. To compute scatter, we aligned the sequences of RFs obtained with CE and IE stimulation (see above) and then computed the distance between RF centers for each pair. This procedure was done separately for the horizontal and vertical axes (which we named horizontal and vertical disparity, respectively; see Fig. 8). Scatter was evaluated by comparing the average distances for RF pairs mapped inside and outside the BS. Typically, scatter is a measure of the dispersion of RF position for electrode penetrations made orthogonal to the cortical surface, i.e., the dispersion of RF position in relation to the average topographical location, within a cortical column. However, we reasoned that, in our case, it would be more appropriate to compare the dispersion of RF position between CE and IE when stimulating inside and outside the BS. This is because only the IE, but not the CE, sends direct efferents to the BSR in V1. The IE would thus provide the BSR with the “standard” topographic organization along with the intrinsic scatter expected to be found throughout V1. Thus the evaluated scatter outside the BSR is the compound measure of two standard scatters, whereas the one inside the BSR is the compound measure of the standard scatter and the scatter we want to assess. In this way, we could estimate the topographic precision of the completion processes taking place inside the BSR and compare it with the rest of V1 (i.e., RFs near the BS).

#### Ocular dominance.

We used an ocular dominance index (ODI) defined as: ODI = (CE − IE)/(CE + IE), where CE and IE correspond to the mean firing rate within the RF region across the eight stimulus conditions obtained with CE and IE stimulation. Positive and negative values indicate neurons preferentially driven by the CE or IE, respectively. For the specific case where we were recording inside the BSR and stimulating the CE, the ODI was calculated using interpolated RF responses generated by the long moving bars.

#### Orientation and direction selectivity.

Neuronal responses to each of the eight stimulus directions were represented as a polargram, where the magnitude and angle of each vector corresponded, respectively, to the mean activation of the neuron and the direction of motion of the stimulus used. The rate of spontaneous activity, measured during the 700-ms window before stimulus onset, was subtracted from the stimulus-driven responses. The vectorial sum of the eight individual vectors indicated the direction preference and its magnitude for that particular neuron. We computed a direction selectivity index by normalizing the magnitude of the sum vector by the total response magnitude across all conditions. An index of 1 indicated sharply tuned cells, whereas an index of 0 indicated nontuned (i.e., pandirectional) cells.

We used an analogous procedure to compute orientation selectivity. First, we combined responses for the two conditions corresponding to the same axis of motion (i.e., bars with same orientation but opposing directions). The orientation selectivity index (OI) was calculated according to Sato et al. (1996): OI = [(∑R_i_sin(2*O*_i_))^2^ + (∑R_i_cos(2*O*_i_))^2^]^0.5^/∑R_i_, where R_i_ represents the response magnitude to each stimulus orientation, *O*_i_. The resulting orientation index varied between 0 (pandirectional neuron) and 1 (entirely orientation selective neuron).

The *t*-test or Wilcoxon test was used for two-sample comparisons. We used parametric or nonparametric statistical tests to analyze orientation and direction selectivity depending on whether the responses could be described by a Gaussian distribution.

#### Histological procedures.

The animals were deeply anesthetized with sodium pentobarbitone (30 mg/kg) and perfused intracardially with the following sequence of solutions: saline, 2% paraformaldehyde in PBS, 2% paraformaldehyde in PBS supplemented with 2.5% glycerol, PBS supplemented with 5% glycerol, and PBS supplemented with 10% glycerol. Frozen sections (70 μm thick) were cut on a cryostat and mounted on glass slides. Two of the four animals were enucleated at the end of the last recording session to reveal cytochrome oxidase (CytOx)-rich regions in V1 (i.e., ocular dominance columns). Thus alternate sections were designated for Nissl (cresyl violet) and for CytOx enzyme histochemistry, which was performed according to the modified Silverman and Tootell (1987) method. The sections stained with cresyl violet were analyzed on a slide projector and microscope to determine the locations of the electrode penetration tracks. The sections reacted for CytOx histochemistry were used to locate the BSR in the roof of the calcarine fissure.

## RESULTS

We present data from 781 recordings acquired inside and near the BSR in area V1. Recordings were performed using single and multielectrodes placed along the horizontal meridian in the calcarine cortex (10–20° eccentricity) of the anesthetized and paralyzed capuchin monkey (4 animals, 6 hemispheres).

In three of the monkeys, we used single electrodes to obtain 492 recordings from 123 penetrations (number of penetrations for each animal and hemisphere: animal A, 16 and 33 in right and left hemisphere, respectively; animal B, 27 in each hemisphere; animal C, 20 in the left hemisphere). For each penetration, we recorded at two different depths yielding 246 sites, and, at each depth, we recorded the activity of both an isolated neuron (SU) and the activity of a small cluster of approximately two to four neurons (MU). SU and MU data did not significantly differ from each other and were therefore pooled together in our analysis. In animal D, we acquired MU neuronal recordings from 289 sites using a 32-electrode array. These data were acquired at different depths from a single placement of the array in the calcarine cortex.

The local mapping procedure (i.e., small stimuli flashed inside the BS) was used as a control to ascertain that the electrode was positioned inside the BSR in V1. The requirement in this case was that neuronal responses could only be elicited with IE stimulation. The automatic version of the local mapping procedure (see materials and methods) was only used in animal D, for which we also stored the corresponding neuronal data for posterior analysis. In animals A, B, and C, we used a manual version of local mapping, where we stimulated exclusively inside the BS using small squares and small bars (neuronal data not stored), which sufficed to confirm that we were recording inside the BSR. The global mapping procedure (i.e., using elongated moving bars) was used for all recording sites in each of the four animals. By using elongated bars that extended beyond the BS borders, we expected to obtain interpolated neuronal responses inside the BSR in V1 because of previously described completion mechanisms (Fiorani Júnior et al. 1992). Our aim here was to investigate the topographic organization of the interpolated responses inside the BSR.

**Fig. 3.**
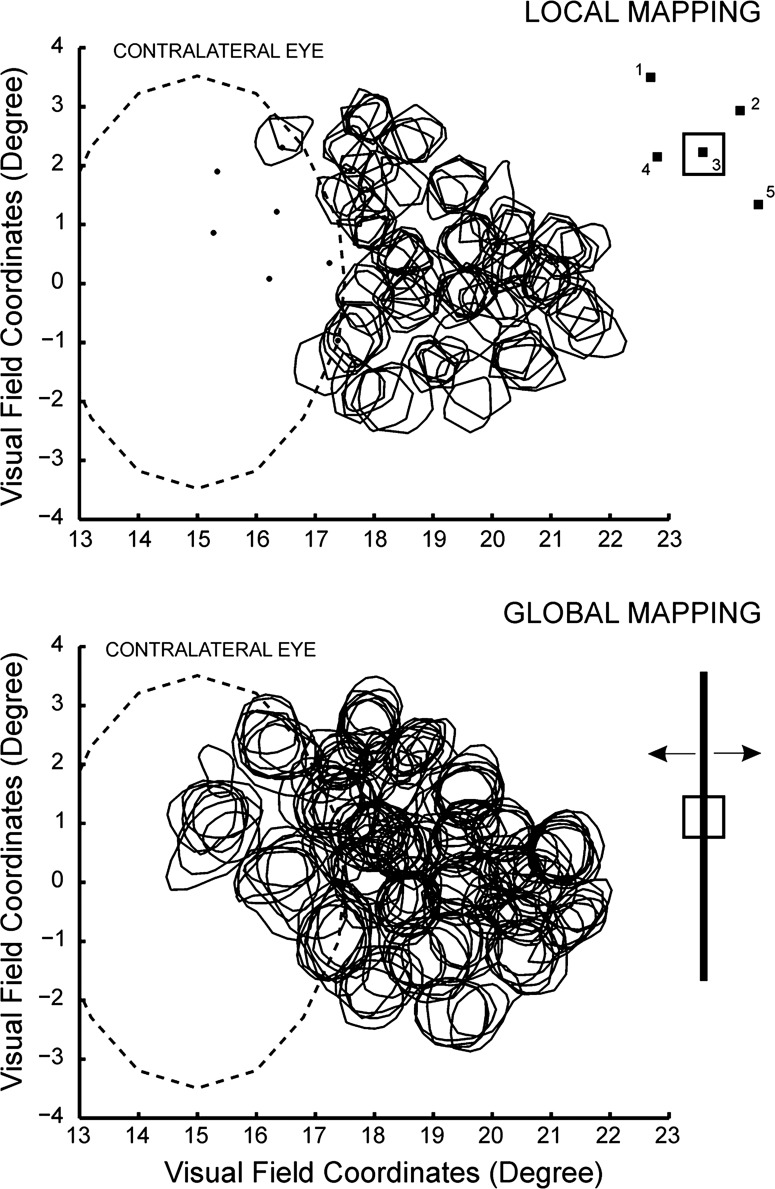
Comparison of the local and global RF mapping methods (recordings performed with the 32 multielectrode array in animal D). RF outlines are represented by the continuous black traces, whereas the dashed oval trace represents the outline of the BS in the visual field. The corresponding visual stimuli used in each case are illustrated on the right (RFs represented by □). The array was positioned in a region of the cortex that partially overlapped the BSR in V1. To investigate the RF organization inside the BSR, we stimulated exclusively through the CE. *Top*: squares flashed inside the BS (i.e., local mapping) failed to yield neuronal responses, confirming the absence of photoreceptor activation (dots predict the average RF centers obtained with IE stimulation). *Bottom*: by stimulating with long moving bars, we were able to elicit V1 activation, and the resulting RFs exhibited highly organized topography.

Figure 3 illustrates data from animal D where we compare the RFs obtained with the automatic local and global mapping procedures carried out with monocular stimulation of the CE. From the 289 sites recorded with the electrode array, we were able to map, respectively, 109 and 181 RFs using the local and global mapping methods. Note that 7 of the 32 electrodes were located inside or on the border of the BSR in V1. For this subset, we were able to map 72 RFs with global mapping and only two with local mapping. Some sites at the border of the BSR showed responses to both methods, with smaller RFs for local mapping.

### 

#### Sequences of RFs mapped through the BS.

**Fig. 4.**
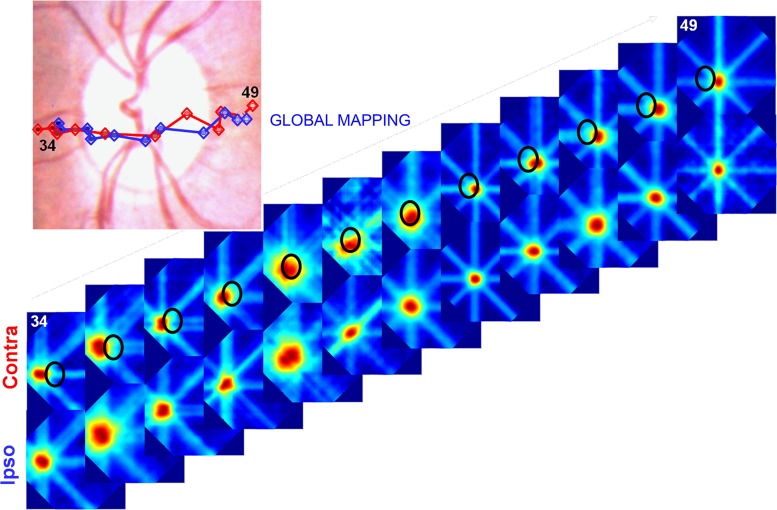
Sequential electrode penetrations that cross the BSR in V1 give rise to an orderly progression of RFs that cross the BS in the visual field. Panels labeled 34 through 49 illustrate the successive single-unit (SU) RFs that were mapped using the global method. They were obtained from 12 single-electrode penetrations placed either inside or in the vicinity of the BSR in V1. Recordings in which the neuronal response did not reach a *z*-score value ≥2 were not included in the sequence. Penetrations were spaced 300 μm apart and followed an approximate linear track. Vertically aligned panels correspond to spiking activity from the same recording obtained with monocular visual stimulation of the CE (Contra) and IE (Ipso), respectively. The BS is represented by the oval shape plotted on the top sequence. *Top*, *left*: sequence of RF centers obtained for the 2 eyes were superimposed on an image of the funds of the eye, color-coded for stimulated eye (red, CE; blue, IE).

Comparison of the RFs mapped with IE and CE stimulation allowed us to evaluate the topographic precision of the BSR. We worked with the assumption that the topography of the BSR when mapped through the IE would be equivalent to the topography found elsewhere in V1. We thereby used it as a reference map when evaluating the topographic organization of the BSR with CE stimulation. To elicit neuronal responses inside the BSR using CE stimulation, we used elongated bars (global mapping method). Sequences of SU RFs obtained with single-electrode recordings for IE and CE stimulation are illustrated in Fig. 4. The RFs were acquired from 12 electrode penetrations that crossed the BSR roughly parallel to the horizontal meridian representation. We mapped the RFs along a linear track of electrode penetrations starting before the BRS, along its linear trajectory through the BSR, and after it exited this region. Vertically aligned panels shown in the upper and lower sequences of Fig. 4 correspond to spiking activity from the same recording but obtained with visual stimulation shown monocularly to each eye in turn. There was a gradual progression of RF centers from the left to the right of the visual field, as expected from the corresponding sequence of electrode penetrations in V1. Notably, the RF centers could be mapped to the inside of the BSR (Fig. 4, *top*), where photoreceptors are known to be absent in the retina. Figure 4, *bottom*, illustrates the corresponding RF sequence resulting from visual stimulation to the IE, where the same stimulus is now capable of directly activating photoreceptors (i.e., the BS in each eye covers different and nonoverlapping portions of the visual field). Importantly, however, the RF sequence obtained for the CE was surprisingly similar to the sequence obtained for the IE, suggesting an elaborate underlying completion mechanism for visual responses in the BSR. The corresponding trajectories of the RF center progression displayed in the *top* and *bottom* rows of Fig. 4 were plotted onto an image of the fundus of the eye (*top*, *left*). Although CE stimulation was not able to directly activate photoreceptors in the retina, both sequences of RF centers are strikingly similar to each other (compare blue and red traces, corresponding to monocular stimulation of the CE or IE, respectively).

**Fig. 5.**
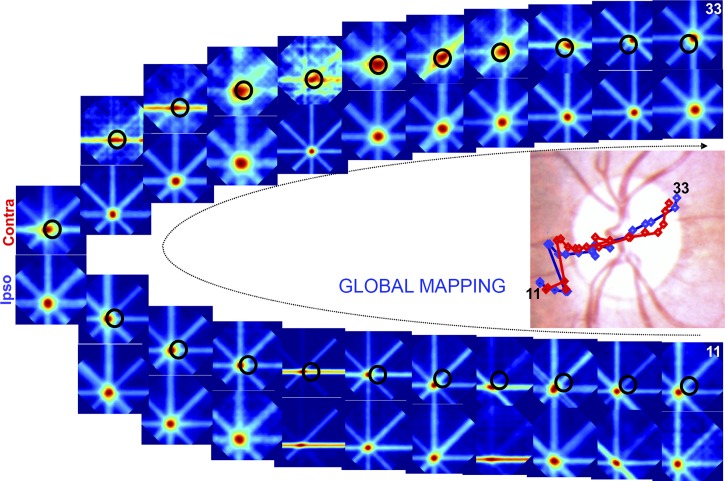
Similar experiment, analysis, and conventions as those shown in Fig 4. Here, the results illustrate multiunit (MU) data from a larger number of electrode penetrations that cross the BSR in a slightly oblique angle (total of 21 penetrations, spaced 300 μm apart). Penetrations followed an approximate linear track in the cortex, except for the sharp transition between penetrations 5 and 6, where we readjusted electrode trajectory to better access the BSR. The resulting sequence of mapped RFs (progression of electrode penetrations indicated by the thin curved arrow) confirms the conclusions drawn from the previous figure.

Figure 5 illustrates a similar experiment and analysis to the one shown in Fig. 4 (same animal and hemisphere, different day) but with a larger number of electrode penetrations (*n* = 21). As before, the RF maps obtained for CE and IE stimulation are strikingly similar to each other, suggesting that a topographic organization of the visual field (i.e., a visuotopic representation) is preserved in the BSR of area V1, regardless of the underlying distribution of photoreceptors in the retina. Note that some of the mapped RFs exhibit elongations in one particular axis (e.g., see Fig. 5 contra eye, 2nd and 3rd panels from the left, RFs mapped inside the BSR). We would like to point out that this does not indicate any abnormality of RFs mapped inside the BSR because the same phenomenon could also be observed for RFs mapped outside of the BSR (e.g., 4th panel from the right, bottom, ipso eye). Elongated RFs, as the ones indicated, arise when the corresponding neurons exhibit strong selectivity for one particular orientation (see Fiorani et al. 2014 for details on the global mapping method). In this case, weak neuronal activity for the axis orthogonal to the preferred orientation hinders a clear delimitation of the corresponding RF border, resulting in the elongated appearance mentioned above.

**Fig. 6.**
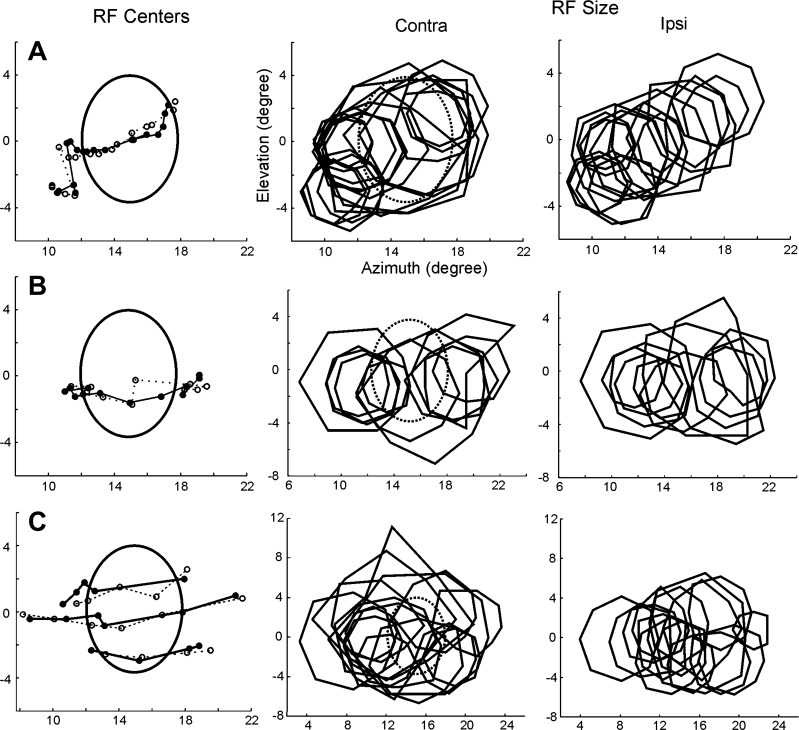
Completion mechanisms taking place inside the BSR give rise to comparatively large RFs. The global mapping method was used for visual stimulation. *A–C*: SU data from animals A, B, and C, respectively, which depict RF center (*left*) and RF size (*middle* and *right*) obtained for electrode penetrations crossing the BSR in V1 (*A*, same experiment as illustrated in Fig 5). ○ and ●, RF centers mapped with CE and IE stimulation, respectively, where continuous and dashed lines connect data points obtained from adjacent electrode penetrations. Oval shapes represent the BS borders in the visual field. Polygons depict RF borders (and hence RF size) mapped through the CE and IE. Note that RFs mapped inside the BS are slightly larger when obtained with CE stimulation compared with IE stimulation. C: 3 sequences of penetrations that probed the BSR at different elevations.

**Fig. 7.**
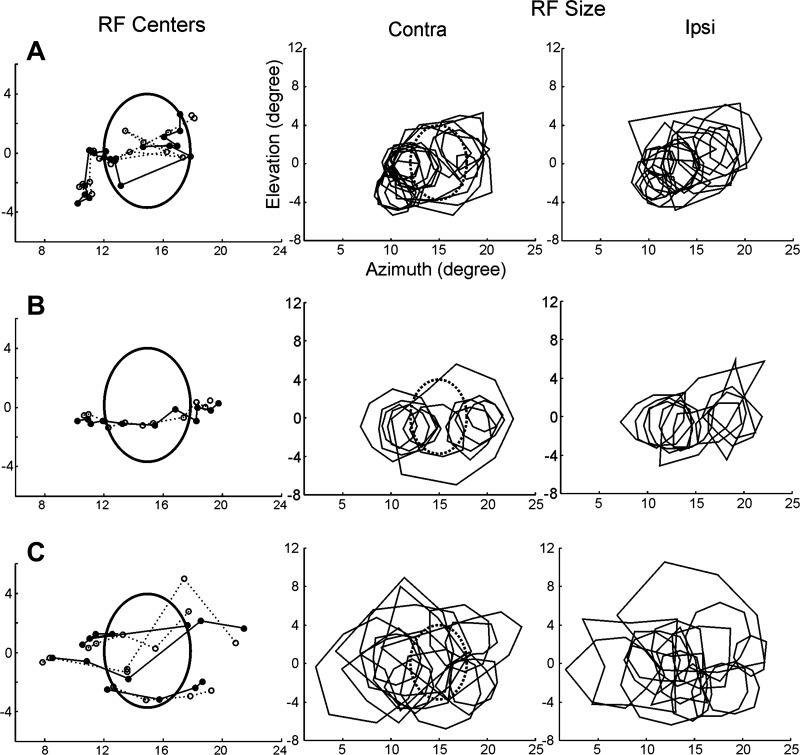
Same set of experiments as the ones shown in Fig 6, but illustrating MU results from neurons isolated from the same corresponding recording sites as in the previous figure. Conventions as before.

After verifying that the completion mechanisms taking place in the BSR are capable of preserving a fine topographic organization, as measured by RF position (Figs. 4 and 5), we sought to investigate whether they also preserved the total area in the visual field to which the neuron responded. To this aim, we compared the RF sizes for recordings made inside and outside the BSR. Specifically, we investigated the sizes of RFs in cases where no direct visual input was available for the BSR (CE stimulation) and compared it to the case where visual afferents were present (IE stimulation). Figures 6 and 7 show results from three experiments in animals A, B, and C, where corresponding panels in both figures illustrate data from different neurons recorded from the same site. Note that RFs are slightly larger when mapped with CE stimulation compared with IE stimulation (we quantify these results below).

In Figs. 6*C* and 7*C*, the RF sequences followed three different isoelevation lines, where the middle line approximately matched the horizontal meridian representation. RF positions in Fig. 7, *A* and *C*, exhibited higher spatial scatter compared with those of Fig. 6 although we can still verify a rather smooth progression of RF positions through the BS. As shown above, sequential electrode penetrations made across the BSR resulted in sequences of RF maps that were contiguous in visual space. Importantly, for all of the three animals studied, the RFs mapped using visual stimulation of the CE were generally larger than the corresponding RFs mapped using IE stimulation, particularly when their centers were located inside the BSR.

Taken together, these results suggest that the BSR in V1 exhibits a topographic organization similar to that found for the rest of V1. These results are trivial when considering only visual stimulation to the IE (Figs. 6 and 7, *right*). However, with monocular stimulation to the CE (Figs. 6 and 7, *middle*), no visual information reaching the BS is transmitted to V1. It is thus intriguing that electrode penetrations across the BSR produced such similar sequences of RF maps for monocular stimulation of the CE and IE (Figs. 6 and 7, *left*), suggesting that the collinear interpolation of neuronal activity elicited in regions surrounding the BSR is generating the precise topographic responses observed within the BSR of V1.

#### Scatter analysis.

To evaluate quantitatively the visual topography in V1, we computed a modified version of RF scatter, where we compare sequences of RFs obtained from the same recording mapped separately for each eye. Specifically, we first aligned the RFs as described in materials and methods, computed the distance (in degrees) for each pair of RFs mapped with CE and IE stimulation, and then compared the average distance for all pairs mapped inside vs. outside the BS. This procedure was done separately for the horizontal and vertical axes in the visual field, which we named horizontal and vertical binocular disparity, respectively.

**Fig. 8.**
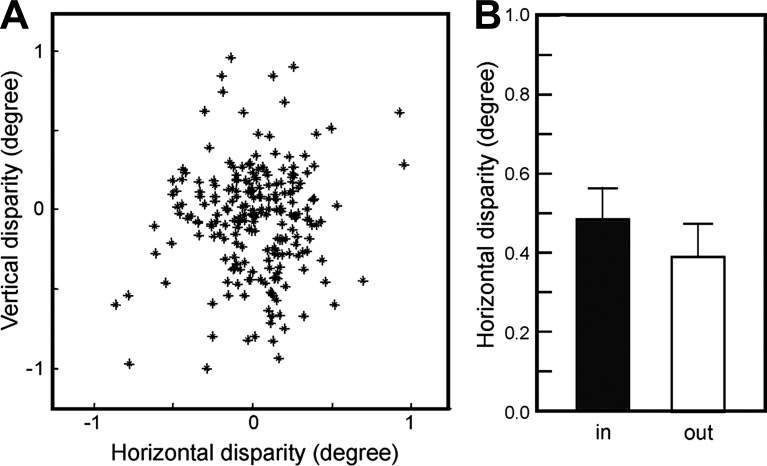
The precision of the topographic organization inside the BSR is similar to the one found elsewhere in V1. *A*: horizontal vs. vertical disparities of RF centers obtained with the global mapping method (*n* = 151). Disparity was computed by subtracting the position of RF centers mapped with each eye (see text for details). *B*: comparison of the mean absolute horizontal disparities for RF centers inside and outside the BS (error bars indicate the standard deviation). No statistical difference was observed between the means of the 2 groups (*P* > 0.05).

We reasoned that, if the BSR is homogeneous and the interpolated RFs mapped with CE stimulation match the topography of the ipsilateral projection, then the magnitude of the disparity in this region should be similar to those of other V1 regions. Figure 8*A* shows population results comparing horizontal vs. vertical disparity for the mapped RFs using the global mapping procedure. Note that the data points on both axes have both negative and positive values, reflecting the relative positions of the RFs mapped with each eye. The distribution of data points, however, center around the 0 value, indicating that there is no systematic spatial bias depending on the eye used for stimulation. Because most of the sequences were recorded along the isoelevation domain (i.e., parallel to the horizontal meridian), we decided to quantify scatter along one dimension, namely the horizontal one. Figure 8*B* depicts the mean absolute horizontal scatter for RFs mapped inside and outside of the BS. Despite the fact that disparity was slightly lower outside compared with the inside of the BS, the difference did not reach statistical significance (*P* > 0.05), suggesting that the precision of visual topography inside the BSR is comparable to the one found outside the BSR. This quantitative evidence indicates that completion mechanisms in V1 are capable of maintaining a precise topographic representation of the visual world, even for regions where photoreceptor information is absent.

#### RF size analysis.

**Fig. 9.**
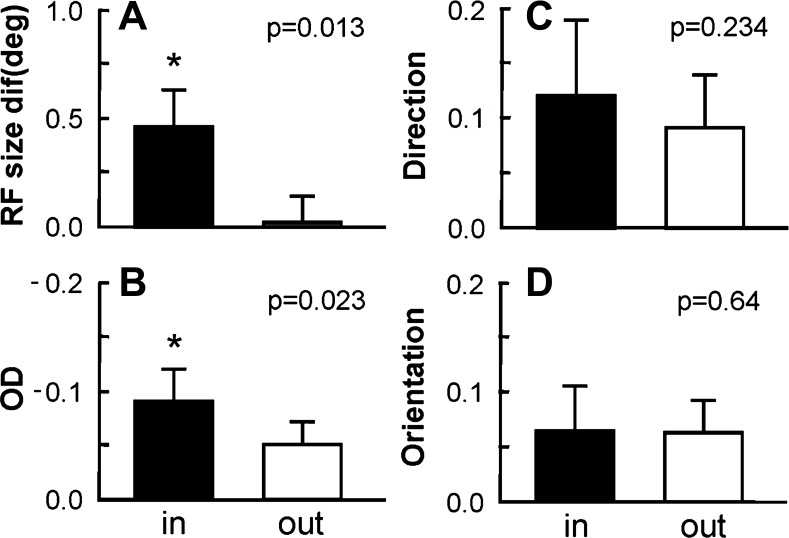
RFs mapped inside and outside the BS differed in properties such as size and ocular dominance (OD) but not in their selectivity for direction and orientation. Data shown are based on the difference between CE and IE measurements for each corresponding variable, calculated separately for RFs mapped inside (solid bars) and outside (open bars) the BS. The global mapping method was used for visual stimulation. *A*: RF sizes were significantly larger when mapped inside the BS, compared with those outside. *B*: neurons recorded inside the BSR responded preferentially to IE stimulation. Note the negative scale for OD on the ordinate of the graph indicating a preponderance of IE dominance. However, no significant differences were observed for either direction (*C*) or orientation selectivity (*D*). *Significant difference (*t*-test, *P* < 0.05).

The results shown in Figs. 6 and 7 suggest differences in RF size for recordings in the BSR, depending on which eye was stimulated. Namely, we noted increased RF sizes for sites recorded within the BSR when stimulating the CE. We hypothesized that the observed increase might be a consequence of integrating visual information across larger portions of the visual field attributable to the lack of photoreceptors in the optic disk region. We therefore decided to quantify the differences in RF sizes for regions inside vs. outside the BS obtained with the global mapping method. The bar graphs in Fig. 9*A* illustrate the average RF sizes for both populations. Indeed, RFs mapped inside the BS were on average larger than those mapped outside (solid and open bars, respectively). The nonparametric Wilcoxon test confirmed a significant difference between the two groups (*P* < 0.05). Size therefore constitutes a major difference between RFs mapped inside vs. outside a major scotoma (i.e., the BS). This result corroborates the more general claim that completion mechanisms give rise to RFs with larger sizes compared with those generated via the classical bottom-up mechanisms.

#### Ocular dominance.

The formula we used to compute ocular dominance generates positive values for neuronal responses dominated by the CE and negative values for those dominated by the IE (see materials and methods). Figure 9*B* shows that recordings carried out inside the BSR responded preferentially to visual stimulation of the IE, as evidenced by their higher negative ODI values. This result was expected because only the IE sends direct retinal input to the BSR.

#### Direction and orientation selectivity.

In primates, area V1 is the first region in the visual hierarchy to exhibit a high prevalence of cells with orientation or direction selectivity (Hubel and Wiesel 1968). Both these properties are the result of intricate neural mechanisms that involve both short- and long-range neuronal interactions responsible for shaping and fine tuning the selectivity of cell responses (Bosking et al. 1997). We worked with the assumption that the completion phenomena taking place in the BSR are the result of long-range neuronal interactions between cortical columns exhibiting similar response properties. In this scenario, visual stimulation using a long-orientated bar moving across the BS would activate modules on the surroundings of the BSR that exhibit similar orientation/direction selectivity. Axons originating from these corresponding modules would thereby converge onto neurons located inside the BSR and be ultimately responsible for the interpolation phenomena we observe. If this were true, we would expect neurons inside the BSR to exhibit similar orientation/direction selectivity regardless of the stimulated eye (i.e., regardless of whether long-range interpolation mechanisms are taking place).

To compare the direction selectivity inside and outside the BSR, we computed the difference in the directionality indices for responses obtained with CE and IE stimulation. Indeed, we found no significant difference between the two groups (Fig. 9*C*, *P* > 0.05). The same analysis was also applied to orientation selectivity, where we obtained similar results (Fig. 9*D*).

## DISCUSSION

Here we investigate the topographic organization of the BSR in monkey area V1 using unbiased, automatic RF mapping methods (Fiorani et al. 2014). The BSR in V1 corresponds to the optic disk region of the CE. Despite the fact that the optic disk is devoid of photoreceptors, neurons in the BSR are nevertheless capable of responding to visual stimulation by means of previously described completion mechanisms (De Weerd et al. 1995; Fiorani Júnior et al. 1992; Gilbert and Wiesel 1992; Kapadia et al. 1995, 1999; Komatsu et al. 1996, 2000; Pettet and Gilbert 1992; Polat et al. 1998). The results we present here demonstrate that these completion mechanisms are capable of interpolating visual information from the surround region of the BS to generate a precise topographic map of the visual world within the BSR. We thereby challenge the currently held belief that area V1 contains a point-by-point representation of the retina (i.e., a retinotopic map; see Blasdel and Campbell 2001). We found instead that V1 represents the world in a continuous fashion, without the interruptions predicted by the distribution of scotomas in the retina. We hereby propose the term “visuotopy” as a more adequate description of how the visual field is represented in V1.

The terms retinotopy and visuotopy have been used interchangeably to describe how topographically organized cortical areas represent the visual world. However, treating both concepts as synonymous can been problematic. For example, stereopsis can be generated by comparing the disparity of visual information across retinas (Pettigrew et al. 1968). Although the information carried by the optic nerve contains a close correspondence with the two-dimensional arrangement of photoreceptors in the retina (i.e., a retinotopic arrangement), this concept does not fully describe the three-dimensional cortical representation that emerges from two-dimensional retinal images. Similarly, the investigation of natural scotomas such as the BS may offer insight into the nature of cortical representation. Here, the concept of retinotopic representation would predict that we perceive a “hole” in the visual field when monocularly viewing the world, something that obviously does not happen. As an alternative, it has been proposed that the brain is simply “unaware” of the absence of visual information in the BS region, an argument with important implications for perception (Andrews and Campbell 1991; Dennett 1992; Dennett and Kinsbourne 1995; Durgin et al. 1995). Previous results from our laboratory and others, however, suggest that is not the case (Churchland and Ramachandran 1996; Fiorani Júnior et al. 1992; Komatsu et al. 2000). Our initial description of the completion phenomenon taking place in V1 opened up the possibility that neuronal activity in the surround region of the BSR could “passively” infiltrate the BSR (Fiorani Júnior et al. 1992). This is because a bar that stimulated a single side of the BS was sometimes capable of eliciting weak neuronal activity inside the BSR. Although these results indicate that the brain does not simply “ignore” the BS, it also does not implicate any relevant role for the BSR in perception because, in this scenario, it would be passively incorporating input from its surround region to fill up a perceptual gap (Awater et al. 2005). Our present data allow us to make a stronger claim. We observed that visual input presented to the BS surround does not passively infiltrate the BSR but that it is used in a coherent manner to generate a precise topographic representation of the visual field inside the BSR, lending support to an active underlying completion mechanism. Indeed, psychophysical studies have shown that the completed image is not perceived as curled at the edges of the BS (Tripathy et al. 1995, 1996), suggesting that completion mechanisms give rise to highly coherent percepts without significant distortions (Das and Gilbert 1997, Letelier and Varela 1984, Schwartz et al. 1985).

In our analysis, we took advantage of the fact that the BSR receives direct visual input from the IE. Therefore, for each recording, we were able to map two RFs, one based on completion mechanisms (CE stimulation) and the other based on direct retinal input (IE stimulation). The later served as a reference map to which we compared the corresponding RFs obtained through completion mechanisms. Because both maps were acquired from the same recording site, we were able to reduce the variance inherent to when comparing activity across sites (e.g., such as when comparing RF maps obtained solely through the CE with stimulation inside and outside the BSR). Note that our underlying assumption here was that the topographic organization of the BSR, as mapped with the IE, is comparable to the one found elsewhere in V1.

During our recordings, we carried out sequences of penetrations that were close enough to each other (∼300 μm apart) to allow for a dense sampling of the BSR and its surroundings. Additionally, we were careful regarding the path and spacing between penetrations to obtain a systematic and homogeneous sampling of the cortical surface. Unfortunately, sequential single-electrode penetrations (as performed in 3 of the 4 animals studied) do not guarantee a homogeneous sampling grid. Thus we were not able, for example, to compute the cortical magnification factor inside the BSR and compare it with the rest of V1. However, it is important to emphasize that homogeneous sampling is not central to the conclusions drawn here. As mentioned above, for each penetration inside the BSR, we were able to obtain an RF map based on completion mechanisms, as well as one based on direct retinal input. This allowed us to assess the precision of the topographic organization inside the BSR regardless of the configuration of electrode penetrations. Despite the close electrode penetrations, our sampling was still not dense enough to allow us to assess the functional organization of direction and orientation columns in the BSR and compare it with other V1 regions.

Our current results show that basic V1 neuronal properties, namely the degree of direction and orientation selectivity, seemed to be preserved for neurons inside the BSR regardless of whether their RFs were mapped through completion mechanisms or direct visual input. Therefore, input from the IE to the BSR can be envisioned as a scaffold upon which completion processes for the CE are carried out. This result corroborates our working hypothesis that long-range collinear interactions in V1 constitute the anatomical substrate for the completion phenomena we observe. V1 horizontal connections, for example, give rise to specialized circuits, which shape the direction and orientation tuning of the neurons by linking columns with similar response properties (Amorim and Picanco-Diniz 1997, Bosking et al. 1997; Buzas et al. 1998, 2001; Crook et al. 1996, 1997, 1998; de Amorim and Picanco-Diniz 1996, Kisvarday et al. 1997, 2000; Sincich and Blasdel 2001). In this way, neurons inside the BSR would receive long-distance input (up to ∼8 mm, see Calford et al. 2003; Chino et al. 1995; Gilbert et al. 1996) from neurons with similar response properties located outside the BSR, explaining the relevance of collinear flanks to the active completion process.

With our present data, we are not able to rule out the participation of feedback projections on the completion phenomenon we describe. Some neurophysiological studies have implicated higher cortical areas in perceptual filling in, a process that is closely related to completion (De Weerd et al. 1995; Matsumoto and Komatsu 2005). Anatomically, feedback projections are highly divergent (Angelucci and Bressloff 2006; Gattass et al. 2005) and connect sites with broader selectivities compared with the intrinsic V1 horizontal connections (Shmuel et al. 2005). Matsumoto and Komatsu (2005) suggested that filling in the BSR might involve feedback signals from extrastriate areas to V1 based on the finding that the average V1 response latency was slower for stimuli presented to the CE compared with the IE. The longer latencies would thereby reflect the time required for feedforward signals to reach higher areas, such as V2, and then feedback to the BSR in V1. We performed a similar analysis for response latency, but their wide distribution of values (∼60–200 ms) did not yield any insight into this issue (data not shown). Future studies that address the differential contributions of horizontal vs. feedback connections to the completion mechanisms in V1 may need to resort to cooling or other inactivation techniques of extrastriate regions.

The highlight of this study was to assess the topographic precision of the BSR using a quantitative, unbiased approach. Notably, we observed that the early visual system employs intricate mechanisms to preserve a continuous and precise representation of the world, regardless of the underlying photoreceptor distribution or the presence of scotomas in the retina. Accordingly, it is curious that we continue to use the term “receptive field” to describe the region of space to which neurons in the BSR respond because, in this case, there are no receptors in the BS that are being directly stimulated.

## GRANTS

The work was funded by grants from PRONEX, CNPq, FUJB, FAPERJ, FINEP, and CEPG. J. C. B. Azzi received a CNPq fellowship for graduate study. R. Gattass is a CNPq Research Fellow.

## DISCLOSURES

No conflicts of interest, financial or otherwise, are declared by the authors.

## AUTHOR CONTRIBUTIONS

Author contributions: J.C.B.A., R.G., and M.F. conception and design of research; J.C.B.A., J.G.M.S., and M.F. performed experiments; J.C.B.A., B.L., and M.F. analyzed data; J.C.B.A. drafted manuscript; R.G., B.L., and M.F. interpreted results of experiments; R.G. prepared figures; R.G., B.L., J.G.M.S., and M.F. edited and revised manuscript; R.G., B.L., J.G.M.S., and M.F. approved final version of manuscript.

## References

[B1] Adams DL, Horton JC Shadows cast by retinal blood vessels mapped in primary visual cortex. Science 298: 572–576, 2002. 12386328 10.1126/science.1074887 PMC3155987

[B2] Adams DL, Horton JC A precise retinotopic map of primate striate cortex generated from the representation of angioscotomas. J Neurosci 23: 3771–3789, 2003a. 12736348 10.1523/JNEUROSCI.23-09-03771.2003 PMC6742198

[B3] Adams DL, Horton JC The representation of retinal blood vessels in primate striate cortex. J Neurosci 23: 5984–5997, 2003b. 12853416 10.1523/JNEUROSCI.23-14-05984.2003 PMC6740354

[B4] Adams DL, Sincich LC, Horton JC Complete pattern of ocular dominance columns in human primary visual cortex. J Neurosci 27: 10391–10403, 2007. 17898211 10.1523/JNEUROSCI.2923-07.2007 PMC6673158

[B5] Amorim AK, Picanco-Diniz CW Horizontal projections of area 17 in Cebus monkeys: metric features, and modular and laminar distribution. Braz J Med Biol Res 30: 1489–1501, 1997. 9686172 10.1590/s0100-879x1997001200018

[B6] Andrews PR, Campbell FW Images at the blind spot. Nature 353: 308, 1991. 1820766 10.1038/353308a0

[B7] Angelucci A, Bressloff PC Contribution of feedforward, lateral and feedback connections to the classical receptive field center and extra-classical receptive field surround of primate V1 neurons. Prog Brain Res 154: 93–120, 2006. 17010705 10.1016/S0079-6123(06)54005-1

[B8] Awater H, Kerlin JR, Evans KK, Tong F Cortical representation of space around the blind spot. J Neurophysiol 94: 3314–3324, 2005. 16033933 10.1152/jn.01330.2004 PMC1401501

[B9] Azzi JCB, Gattass R, Fiorani M Visuotopic organization of the blind spot representation in V1 in primates. Program No. 164.9. 2000 Neuroscience Meeting Planner. New Orleans, LA: Society for Neuroscience, 2000.

[B10] Blasdel G, Campbell D Functional retinotopy of monkey visual cortex. J Neurosci 21: 8286–8301, 2001. 11588200 10.1523/JNEUROSCI.21-20-08286.2001 PMC6763878

[B11] Bosking WH, Zhang Y, Schofield B, Fitzpatrick D Orientation selectivity and the arrangement of horizontal connections in tree shrew striate cortex. J Neurosci 17: 2112–2127, 1997. 9045738 10.1523/JNEUROSCI.17-06-02112.1997 PMC6793759

[B12] Botelho EP, Ceriatte C, Soares JG, Gattass R, Fiorani M Quantification of early stages of cortical reorganization of the topographic map of V1 following retinal lesions in monkeys. Cereb Cortex 24: 1–16, 2014. 23010747 10.1093/cercor/bhs208 PMC3862261

[B13] Buzas P, Eysel UT, Kisvarday ZF Functional topography of single cortical cells: an intracellular approach combined with optical imaging. Brain Res Brain Res Protoc 3: 199–208, 1998. 9813324 10.1016/s1385-299x(98)00041-5

[B14] Buzas P, Eysel UT, Adorjan P, Kisvarday ZF Axonal topography of cortical basket cells in relation to orientation, direction, and ocular dominance maps. J Comp Neurol 437: 259–285, 2001. 11494255 10.1002/cne.1282

[B15] Calford MB, Wright LL, Metha AB, Taglianetti V Topographic plasticity in primary visual cortex is mediated by local corticocortical connections. J Neurosci 23: 6434–6442, 2003. 12878683 10.1523/JNEUROSCI.23-16-06434.2003 PMC6740630

[B16] Chino YM, Smith EL 3rd, Kaas JH, Sasaki Y, Cheng H Receptive-field properties of deafferentated visual cortical neurons after topographic map reorganization in adult cats. J Neurosci 15: 2417–2433, 1995. 7891177 10.1523/JNEUROSCI.15-03-02417.1995 PMC6578169

[B17] Churchland PS, Ramachandran VS Why Dennett is wrong. In: Perception, edited by Kathleen Akins Oxford, UK: Oxford University, 1996, pp. 132–157.

[B18] Crook JM, Kisvarday ZF, Eysel UT GABA-induced inactivation of functionally characterized sites in cat visual cortex (area 18): effects on direction selectivity. J Neurophysiol 75: 2071–2088, 1996. 8734604 10.1152/jn.1996.75.5.2071

[B19] Crook JM, Kisvarday ZF, Eysel UT GABA-induced inactivation of functionally characterized sites in cat striate cortex: effects on orientation tuning and direction selectivity. Vis Neurosci 14: 141–158, 1997. 9057276 10.1017/s095252380000883x

[B20] Crook JM, Kisvarday ZF, Eysel UT Evidence for a contribution of lateral inhibition to orientation tuning and direction selectivity in cat visual cortex: reversible inactivation of functionally characterized sites combined with neuroanatomical tracing techniques. Eur J Neurosci 10: 2056–2075, 1998. 9753093 10.1046/j.1460-9568.1998.00218.x

[B21] Das A, Gilbert CD Distortions of visuotopic map match orientation singularities in primary visual cortex. Nature 387: 594–598, 1997. 9177346 10.1038/42461

[B22] de Amorim AK, Picanço-Diniz CW Intrinsic projections of Cebus-monkey area 17: cell morphology and axon terminals. Rev Bras Biol 56, Suppl 1: 209–219, 1996. 9394502

[B23] Dennett D Consciousness Explained. New York, NY: Back Bay Books, 1992.

[B24] Dennett DC, Kinsbourne M Time and the observer: The where and when of consciousness in the brain. Behav Brain Sci 15: 183–201, 1995.

[B25] De Weerd P, Gattass R, Desimone R, Ungerleider LG Responses of cells in monkey visual cortex during perceptual filling-in of an artificial scotoma. Nature 377: 731–734, 1995. 7477262 10.1038/377731a0

[B26] Dow BM, Snyder AZ, Vautin RG, Bauer R Magnification factor and receptive field size in foveal striate cortex of the monkey. Exp Brain Res 44: 213–228, 1981. 7286109 10.1007/BF00237343

[B27] Durgin FH, Tripathy SP, Levi DM On the filling in of the visual blind spot: some rules of thumb. Perception 24: 827–840, 1995. 8710443 10.1068/p240827

[B28] Fiorani M, Azzi JC, Soares JG, Gattass R Automatic mapping of visual cortex receptive fields: a fast and precise algorithm. J Neurosci Methods 221: 112–126, 2014. 24084390 10.1016/j.jneumeth.2013.09.012

[B29] Fiorani Júnior M, Rosa MG, Gattass R, Rocha-Miranda CE Dynamic surrounds of receptive fields in primate striate cortex: a physiological basis for perceptual completion? Proc Natl Acad Sci USA 89: 8547–8551, 1992. 1528860 10.1073/pnas.89.18.8547 PMC49957

[B30] Gattass R, Sousa AP, Rosa MG Visual topography of V1 in the Cebus monkey. J Comp Neurol 259: 529–548, 1987. 3597827 10.1002/cne.902590404

[B31] Gattass R, Pessoa LA, de Weerd P, Fiorani M Filling-in in topographically organized distributed networks. An Acad Bras Cienc 71: 997–1015, 1999. 10683675

[B32] Gattass R, Nascimento-Silva S, Soares JG, Lima B, Jansen AK, Diogo AC, Farias MF, Botelho MM, Mariani OS, Azzi J, Fiorani M Cortical visual areas in monkeys: location, topography, connections, columns, plasticity and cortical dynamics. Philos Trans R Soc Lond B Biol Sci 360: 709–731, 2005. 15937009 10.1098/rstb.2005.1629 PMC1569490

[B33] Gilbert CD, Wiesel TN Receptive field dynamics in adult primary visual cortex. Nature 356: 150–152, 1992. 1545866 10.1038/356150a0

[B34] Gilbert CD, Das A, Ito M, Kapadia M, Westheimer G Spatial integration and cortical dynamics. Proc Natl Acad Sci USA 93: 615–622, 1996. 8570604 10.1073/pnas.93.2.615 PMC40100

[B35] Hochstein S, Ahissar M View from the top: hierarchies and reverse hierarchies in the visual system. Neuron 36: 791–804, 2002. 12467584 10.1016/s0896-6273(02)01091-7

[B36] Hubel DH, Wiesel TN Receptive fields and functional architecture of monkey striate cortex. J Physiol 195: 215–243, 1968. 4966457 10.1113/jphysiol.1968.sp008455 PMC1557912

[B37] Hubel DH, Wiesel TN Sequence regularity and geometry of orientation columns in the monkey striate cortex. J Comp Neurol 158: 267–293, 1974. 4436456 10.1002/cne.901580304

[B38] Jansen-Amorim AK, Lima B, Fiorani M, Gattass R GABA inactivation of visual area MT modifies the responsiveness and direction selectivity of V2 neurons in Cebus monkeys. Vis Neurosci 28: 513–527, 2011. 22192507 10.1017/S0952523811000411

[B39] Jansen-Amorim AK, Fiorani M, Gattass R GABA inactivation of area V4 changes receptive-field properties of V2 neurons in Cebus monkeys. Exp Neurol 235: 553–562, 2012. 22465265 10.1016/j.expneurol.2012.03.008

[B40] Kapadia MK, Ito M, Gilbert CD, Westheimer G Improvement in visual sensitivity by changes in local context: parallel studies in human observers and in V1 of alert monkeys. Neuron 15: 843–856, 1995. 7576633 10.1016/0896-6273(95)90175-2

[B41] Kapadia MK, Westheimer G, Gilbert CD Dynamics of spatial summation in primary visual cortex of alert monkeys. Proc Natl Acad Sci USA 96: 12073–12078, 1999. 10518578 10.1073/pnas.96.21.12073 PMC18414

[B42] Kisvarday ZF, Toth E, Rausch M, Eysel UT Orientation-specific relationship between populations of excitatory and inhibitory lateral connections in the visual cortex of the cat. Cereb Cortex 7: 605–618, 1997. 9373017 10.1093/cercor/7.7.605

[B43] Kisvarday ZF, Crook JM, Buzas P, Eysel UT Combined physiological-anatomical approaches to study lateral inhibition. J Neurosci Methods 103: 91–106, 2000. 11074099 10.1016/s0165-0270(00)00299-5

[B44] Komatsu H, Murakami I, Kinoshita M Surface representation in the visual system. Brain Res Cogn Brain Res 5: 97–104, 1996. 9049075 10.1016/s0926-6410(96)00045-6

[B45] Komatsu H, Kinoshita M, Murakami I Neural responses in the retinotopic representation of the blind spot in the macaque V1 to stimuli for perceptual filling-in. J Neurosci 20: 9310–9319, 2000. 11125010 10.1523/JNEUROSCI.20-24-09310.2000 PMC6773023

[B46] Letelier JC, Varela F Why the cortical magnification factor in rhesus is isotropic. Vision Res 24: 1091–1095, 1984. 6506474 10.1016/0042-6989(84)90087-7

[B47] Lin Z, He S Emergent filling in induced by motion integration reveals a high-level mechanism in filling in. Psychol Sci 23: 1534–1541, 2012. 23085642 10.1177/0956797612446348 PMC3875405

[B48] Matsumoto M, Komatsu H Neural responses in the macaque V1 to bar stimuli with various lengths presented on the blind spot. J Neurophysiol 93: 2374–2387, 2005. 15634711 10.1152/jn.00811.2004

[B49] Maunsell JH, Gibson JR Visual response latencies in striate cortex of the macaque monkey. J Neurophysiol 68: 1332–1344, 1992. 1432087 10.1152/jn.1992.68.4.1332

[B50] McGuire PK, Bates JF, Goldman-Rakic PS Interhemispheric integration: I. Symmetry and convergence of the corticocortical connections of the left and the right principal sulcus (PS) and the left and the right supplementary motor area (SMA) in the rhesus monkey. Cereb Cortex 1: 390–407, 1991a. 1726605 10.1093/cercor/1.5.390

[B51] McGuire PK, Bates JF, Goldman-Rakic PS Interhemispheric integration: II. Symmetry and convergence of the corticostriatal projections of the left and the right principal sulcus (PS) and the left and the right supplementary motor area (SMA) of the rhesus monkey. Cereb Cortex 1: 408–417, 1991b. 1726606 10.1093/cercor/1.5.408

[B52] Moratti S, Méndez-Bértolo C, Del-Pozo F, Strange BA Dynamic gamma frequency feedback coupling between higher and lower order visual cortices underlies perceptual completion in humans. Neuroimage 86: 470–479, 2014. 24185019 10.1016/j.neuroimage.2013.10.037

[B53] Pettet MW, Gilbert CD Dynamic changes in receptive-field size in cat primary visual cortex. Proc Natl Acad Sci USA 89: 8366–8370, 1992. 1518870 10.1073/pnas.89.17.8366 PMC49919

[B54] Pettigrew JD, Nikara T, Bishop PO Binocular interaction on single units in cat striate cortex: simultaneous stimulation by single moving slits with receptive fields in correspondence. Exp Brain Res 6: 391–410, 1968. 5721767 10.1007/BF00233186

[B55] Polat U, Mizobe K, Pettet MW, Kasamatsu T, Norcia AM Collinear stimuli regulate visual responses depending on cell's contrast threshold. Nature 391: 580–584, 1998. 9468134 10.1038/35372

[B56] Raiguel SE, Lagae L, Gulyas B, Orban GA Response latencies of visual cells in macaque areas V1, V2 and V5. Brain Res 493: 155–159, 1989. 2776003 10.1016/0006-8993(89)91010-x

[B57] Rosa MG, Gattass R, Fiorani Júnior M Complete pattern of ocular dominance stripes in V1 of a New World monkey, *Cebus apella*. Exp Brain Res 72: 645–648, 1988. 3234508 10.1007/BF00250609

[B58] Rosa MG, Gattass R, Fiorani M Jr, Soares JG Laminar, columnar and topographic aspects of ocular dominance in the primary visual cortex of Cebus monkeys. Exp Brain Res 88: 249–264, 1992. 1577100 10.1007/BF02259100

[B59] Sato H, Katsuyama N, Tamura H, Hata Y, Tsumoto T Mechanisms underlying orientation selectivity of neurons in the primary visual cortex of the macaque. J Physiol 494: 757–771, 1996. 8865072 10.1113/jphysiol.1996.sp021530 PMC1160675

[B60] Schiller PH, Finlay BL, Volman SF Quantitative studies of single-cell properties in monkey striate cortex. I. Spatiotemporal organization of receptive fields. J Neurophysiol 39: 1288–1319, 1976. 825621 10.1152/jn.1976.39.6.1288

[B61] Schwartz E, Tootell RB, Silverman MS, Switkes E, De Valois RL On the mathematical structure of the visuotopic mapping of macaque striate cortex. Science 227: 1065–1066, 1985. 3975604 10.1126/science.3975604

[B62] Shmuel A, Korman M, Sterkin A, Harel M, Ullman S, Malach R, Grinvald A Retinotopic axis specificity and selective clustering of feedback projections from V2 to V1 in the owl monkey. J Neurosci 25: 2117–2131, 2005. 15728852 10.1523/JNEUROSCI.4137-04.2005 PMC6726055

[B63] Silverman MS, Tootell RB Modified technique for cytochrome oxidase histochemistry increased staining intensity and compatibility with 2-deoxyglucose autoradiography. J Neurosci Methods 19: 1–10, 1987. 2434810 10.1016/0165-0270(87)90016-1

[B64] Sincich LC, Blasdel GG Oriented axon projections in primary visual cortex of the monkey. J Neurosci 21: 4416–4426, 2001. 11404428 10.1523/JNEUROSCI.21-12-04416.2001 PMC6762731

[B65] Soares JG, Diogo AC, Fiorani M, Souza AP, Gattass R Effects of inactivation of the lateral pulvinar on response properties of second visual area cells in Cebus monkeys. Clin Exp Pharmacol Physiol 31: 580–590, 2004. 15479164 10.1111/j.1440-1681.2004.04051.x

[B66] Tripathy SP, Levi DM, Ogmen H, Harden C Perceived length across the physiological blind spot. Vis Neurosci 12: 385–402, 1995. 7786858 10.1017/s0952523800008051

[B67] Tripathy SP, Levi DM, Ogmen H Two-dot alignment across the physiological blind spot. Vision Res 36: 1585–1596, 1996. 8759461 10.1016/0042-6989(95)02200-7

